# Incidence of squamous-cell carcinoma of the conjunctiva and other eye cancers in the NIH-AARP Diet and Health Study

**DOI:** 10.3332/ecancer.2012.254

**Published:** 2012-05-21

**Authors:** B Emmanuel, E Ruder, S-W Lin, C Abnet, AR Hollenbeck, SM Mbulaiteye

**Affiliations:** 1 Division of Cancer Epidemiology and Genetics, National Cancer Institute, Bethesda, MD, USA; 2 Department of Sports Medicine and Nutrition, School of Health and Rehabilitation Sciences, University of Pittsburgh, Pittsburgh, PA; 3 AARP, Washington, District of Columbia, USA

## Abstract

**Purpose:**

To investigate the risk factors for squamous-cell carcinoma of the conjunctiva (SCCC) and other eye cancers in the NIH-AARP Diet and Health Study.

**Methods:**

We estimated incidence rates and associations with age, sex, race/ethnicity and ultraviolet radiation using Cox proportional hazards models.

**Results:**

The incidence was 37.3 per 10^6^ for all eye cancers (N = 178), 8.4 per 10^6^ for SCCC (N = 40) and 28.9 per 10^6^ for other eye cancers (N = 138). For all eye cancers, the incidence was lower in women than in men (hazard ratio [HR] = 0.38, 95% CI: 0.26, 0.55) and in persons aged ≤60 years than those aged >60 years (HR = 0.51, 95% CI: 0.36, 0.72). The incidence was higher, but not statistically significant, in those with an average net erythemal exposure >170 versus ≤170 (HR = 1.19, 95% CI: 0.88, 1.63) and lower in those residing at latitudes >35° versus ≤35° (HR = 0.81, 95% CI: 0.61, 1.09). The patterns were similar for SCCC in sex, age, race/ethnicity and average net erythemal exposure, but not statistically significant.

**Conclusion:**

The incidence of all eye cancers was associated with male sex and older age. The same patterns were observed for SCCC. The associations reported here might be surrogates of exposure to ultraviolet radiation, although more follow-up is needed to obtain precise results.

## Background

SCCC is a rare cancer of the ocular surface with an incidence rate that varies geographically from 0.02 per 100,000 in high latitude areas to 3.5 per 100,000 at low latitudes near the Equator [1,2]. This positive correlation between SCCC incidence and latitude indicates that exposure to ultraviolet radiation may play a role in the etiology of SCCC. The risk is higher in males than in females. This may be because men are more likely to have outdoor occupations, which are associated with a greater propensity for exposure to ultraviolet radiation [1,2]. The other known risk of SCCC is immunosuppression, which is associated with 10 times higher SCCC risk in persons due to human immunodeficiency infection (HIV) and post-transplant immunosuppression [3–8]. The association with HIV was first noted in Uganda [3], based on an HIV prevalence of 75% in SCCC patients admitted at a tertiary hospital, compared to an HIV prevalence of 19% in age- and sex-matched controls with other eye conditions attending the same hospital [3], and confirmed in other parts of Africa [3–7] and in the United States [9]. In the United States, SCCC risk in persons with HIV/AIDS was higher in persons aged ≥50 years, of Hispanic ethnicity and who resided in the southern low altitude states [9], but not route of HIV acquisition. Among the general population in the United States, using data from the Surveillance, Epidemiology, and End Results (SEER) Program of the National Cancer Institute, the risk of SCCC was higher in males and in Whites and was correlated to ultraviolet radiation [1]. In the current study, we investigated the risk of SCCC and other eye cancers among healthy, older Americans using data from the National Institutes of Health (NIH)-AARP Diet and Health Study [10], who have a low prevalence of HIV/AIDS, to complement our knowledge of SCCC risk obtained from immunosuppressed persons.

## Methods

### Study population

We used data from the NIH-AARP Diet and Health Study [10]. Briefly, the NIH-AARP cohort comprises 566,401 individuals aged 50–71 years recruited from six US states and two metropolitan areas. Participants provided baseline questionnaire data during 1995–1996 and annual questionnaires update home address and vital status. Individuals whose data were based on proxy respondents and self-reported cancer conditions were excluded from the current analysis for a cohort of 487,770 persons followed up for an average of 9.78 years per individual. Cancer diagnosis was documented through annual record linkage with state cancer registries. Cancer incidence was calculated by dividing the number of cancer cases by the total person-years of follow-up. Person-years of follow-up for each participant was calculated from the date of baseline registration to the date a participant moved out of the catchment area, died, developed eye cancer, or the last date of follow-up (31 December 2006) [10,11].

Eye and orbit cancers were defined according to the International Classification of Diseases (ICD-10) codes C69.0–C69.9 grouped into SCCC (C69.0) and other eye cancers (C69.1–69.9). Ultraviolet radiation exposure was estimated based on the average net erythemal exposure from National Aeronautics and Space Administration (NASA) Total Ozone Mapping Spectrometer (TOMS) according to the residence of the participant at baseline [12]. The NASA TOMS database provides daily information on a noon-time ground-level erythemal estimate on a 1° latitude by 1.25° longitude grid [13]. Each participant was assigned a ground-level erythemal dose by linking the census track centroid of the self-reported residence at baseline, closest to the point on the TOMS grid. Census tract of each participant was assigned by a computer program spatially on longitude and latitude coordinates on geocoding residential addresses. The actual addresses were not available to the investigators. The erythemal dose was averaged across all available measured days in the months of January and July between 1978–1993 and 1996–2005 [12,13]. In addition, state residence and latitude were used to measure ultraviolet radiation based on census tract location where the participant resided at baseline.

Institutional review boards at the National Cancer Institute approved the NIH-AARP Diet and Health Study, and all participants gave written informed consent to participate.

### Statistical analysis

Analyses included all eye cancers, including primary (N = 155) and secondary cancers (N = 23, including 8 that were SCCC). As the secondary eye cancers appeared unrelated to the primary cancers, i.e. a random set of cancers, we decided to combine the primary and secondary eye cancers to improve statistical stability of the results. Our primary interest was to examine risk factors for SCCC, but the numbers were sparse, so we conducted analyses examining risk factors of SCCC, of other eye cancers and of both groups combined. Although it would have been preferable to examine associations across the linear range of variables, the data were somewhat sparse. We thus grouped age as ≤60 years and >60 years, average net erythemal exposure as ≤170 and >170 and latitude as ≤35° and >35° to improve statistical stability of our estimates. The cut-offs for these categories were at medians based on *a priori* decisions to capture maximum contrasts in between the groups. Crude incidence and HR with 95% confidence intervals (CI) of all eye cancers and subgroups were calculated using Cox proportional hazard regression. Incidence was age-adjusted to the US population in 2000 to compare our results to those of the US general population aged 50–74 years. We adjusted associations for age and sex, which have been consistently shown to be linked to SCCC risk. A two-sided P-value <0.05, without adjustment for multiple comparisons, was considered statistically significant. Analysis was performed using SAS version 9.1 (SAS Institute, Cary, NC).

## Results

A total of 178 incident eye cancer cases were identified in the cohort (N = 141 and N = 37 in males and females, respectively) (Table 1). Of these, 155 (87%) were primary eye cancers and 23 were secondary eye cancers. The antecedent cancers for the secondary eye cancers were prostate (N = 9), skin (N = 4) or urinary (N = 3). There was no change in the patterns with the inclusion or exclusion of these cancers. Most cancers were identified in males (79%), in participants aged ≥60 years at baseline (77%) and in non-Hispanic whites (96%). No cancers were noted among Blacks, most likely because this group comprised only 3.9% of the cohort. Slightly over half (57%) of the cancers occurred in participants residing in California, Florida and North Carolina, although these regions comprised 60% of the cohort.

Overall, the incidence of eye cancers was 37.3 per 10^6^. The incidence was lower in women than in men (19.0 versus 50.7 per 10^6^; HR = 0.38, 95% CI: 0.26, 0.55) and lower in participants aged 60 years or younger versus older participants (23.2 versus 46.3 per 10^6^; HR = 0.51, 95% CI: 0.36, 0.72). The incidence was slightly elevated among individuals with an average net erythemal exposure >170 versus ≤170 (HR = 1.22, 95% CI: 0.89, 1.65), slightly decreased in individuals residing at latitude greater than 35° compared to those residing at a latitude less than 35° (HR = 0.81, 95% CI: 0.60, 1.08). The age-adjusted incidence rate for all eye cancers was 7.02 per 10^5^, and it was higher in males (9.73) than in females (3.04).

A total of 40 eye cancers were SCCC (22%) and 138 were other eye cancers (78%) (Table 2). The incidence of SCCC was 8.4 per 10^6^; for other eye cancers, it was 28.9 per 10^6^. The patterns of incidence for SCCC and other eye cancers resembled those observed for all eye cancers combined with respect to sex, age, race/ethnicity, average net erythemal exposure, state of residence and latitude, but the results were not statistically significant for SCCC. For other eye cancers, the incidence was significantly lower in women than in men (13.3 vs. 40.0 per 10^6^; HR = 0.34, 95% CI: 0.22, 0.51) and higher in participants aged ≥60 years at baseline than those who were 60 years or younger (35.6 vs. 17.5 per 10^6^; HR = 0.49, 95% CI: 0.33, 0.73).

## Discussion

Our results are from the largest cohort study to date of mostly immune competent adults in the United States. Even so, the cases were sparse, highlighting the relatively low incidence of SCCC in particular and ocular cancers in general in the United States. Our SCCC findings agree with those reported among immunosuppressed persons with HIV or transplantation indicating that risk of SCCC is higher among males, older individuals and individuals who reside at lower latitudes and experience high erythemal exposures. These associations have been interpreted as indicating a role of greater exposure to ultraviolet radiation in eye cancers, based mostly on studies conducted in Africa [3,4,6,14,15] and to a lesser extent on studies conducted in the United States [9] and in Australia [16,17]. An interesting, although not entirely surprising, finding is that the age-adjusted incidence rate for SCCC is lower in our study than in the comparable US population during 1998–2002. The incidences were 9.73 and 3.04 per 10^5^ for males and females, respectively, in our study and 11.5 and 7.7 per 10^5^ for males and females, respectively, in the United States [18]. The apparent difference in incidence may be an example of a healthy worker or a cohort effect. The NIH-AARP cohort is comprised mostly of individuals from a higher socioeconomic status in the United States whose risk of cancer may be lower than that of the US general population. Alternatively, the NIH AARP cohort may comprise individuals whose average exposure to solar ultraviolet radiation, which is considered the most likely biological risk factor for SCCC [1,19,20] and/or prevalence of immunosuppression due to HIV/AIDS, is lower than the general population.

Our study has limitations, notably including the small number of cases accrued thus far. This limited our statistical power to detect new associations, particularly across the linear range of exposures, and to conduct subgroup analyses. The number of SCCC cases was small, which compelled us to combine primary and secondary cancers to increase statistical stability. This should be considered when interpreting our results. Our study provides no new insights on the interaction between immunosupression and solar ultraviolet radiation in the etiology of SCCC. Understanding the biological basis for the interaction would be useful for prevention, screening and early treatment to reduce morbidity for SCCC in populations at risk. Future work is needed to expand the number of cases available from representative populations with prospective data of high quality, including residence and solar ultraviolet radiation exposure, to obtain sound conclusions about the risk factors of SCCC.

## Conclusions

To conclude, eye cancers including SCCC are rare with incidence associated with males, older age and residing at lower latitude or high erythemal exposures areas complementary to previous studies. These characteristics might be surrogates of exposure to ultraviolet radiation.

## Conflicts of Interest

The author(s) declare that they have no competing interests.

## Author’s Contributions

BE reviewed, analysed and interpreted data. ER, SWL and CA interpreted data. ARH conceived both the AARP cohort and AARP study design; interpreted data. SMM conceived both the study idea and design; reviewed and interpreted data. All authors contributed to writing the manuscript and approved the final draft.

## Figures and Tables

**Table 1:** Characteristics of the cohort population in the NIH-AARP Diet and Health Study and eye cancer cases

	Cohort			Eye Cancer Cases				
Characteristics	Person-years	N	(%)	N	(%)	Crude Incidence (10^6^)	Hazard Ratio (95% CI)	Adjusted Hazard Ratio (95% CI)*
Total	4,769,686	487,700	100	178	100	37.3		
**Sex**								
Male	2,820,412	290,770	59.6	141	79.2	50.7	Reference	Reference
Female	1,949,274	196,930	40.4	37	20.8	19.0	0.38 (0.26, 0.55)	0.38 (0.27, 0.55)
**Age, years**								
≤60	1,768,476	177,517	36.4	41	23.0	23.2	0.51 (0.36, 0.72)	0.51 (0.36, 0.73)
> 60	3,001,210	310,183	63.6	137	77.0	46.3	Reference	Reference
**Race/Ethnicity**								
White (non-Hispanic)	4,342,907	444,272	91.1	170	95.5	39.6	Reference	Reference
Black (non-Hispanic)	188,492	19,204	3.9	0	0.0	N/A	N/A	N/A
Hispanic	93,398	9,375	1.9	4	2.3	42.8	1.10 (0.41, 2.95)	1.1 (0.41, 2.97)
Asian, Pacific Islander or American Indian/Alaska Native	79,996	8,104	1.7	2	1.1	25.0	0.64 (0.16, 2.58)	0.64 (0.16, 2.58)
Unknown	64,893	6,745	1.4	2	1.1	30.8	0.79 (0.20, 3.17)	0.82 (0.20, 3.32)
**Latitude, degree**								
≤ 35	2,156,115	221,493	45.4	90	50.6	42.7	Reference	Reference
> 35	2,613,571	266,207	54.6	88	49.4	33.7	0.81 (0.60, 1.08)	0.81 (0.61, 1.09)
**Average net erythemal exposure**								
≤170	3,312,387	337,596	69.2	116	65.2	35.0	Reference	Reference
> 170	1,457,299	150,104	30.8	62	34.8	43.9	1.22 (0.89, 1.65)	1.19 (0.88, 1.63)
**State of residence**								
California	1,466,258	150,886	30.9	57	32.0	40.4	Reference	Reference
Florida	1,005,607	103,632	21.3	45	25.3	44.7	1.15 (0.78, 1.70)	1.11 (0.75, 1.64)
Georgia	134,585	13,707	2.8	5	2.8	37.2	0.95 (0.38, 2.38)	0.99 (0.40, 2.47)
Louisiana	180,948	18,412	3.8	7	3.9	38.7	0.99 (0.45, 2.17)	1.01 (0.46, 2.22)
Michigan	244,057	24,747	5.1	5	2.8	20.5	0.53 (0.21, 1.31)	0.54 (0.22, 1.35)
North Carolina	395,738	40,200	8.2	16	9.0	40.4	1.04 (0.60, 1.80)	1.02 (0.59, 1.78)
New Jersey	608,434	62,131	12.7	21	11.8	34.5	0.88 (0.54, 1.46)	0.88 (0.53, 1.45)
Pennsylvania	734,059	73,985	15.2	22	12.4	30.0	0.77 (0.47, 1.25)	0.75 (0.46, 1.23)

* Adjusted for sex and age

**Table 2:** Characteristics SCCC and other eye cancers in the cohort population of the NIH-AARP Diet and Health Study

	SCCC					Other Eye Cancers				
Characteristics	N	(%)	Crude Incidence (10^6^)	Hazard Ratio (95% CI)	Adjusted Hazard Ratio (95% CI)*	N	(%)	Crude Incidence (10^6^)	Hazard Ratio (95% CI)	Adjusted Hazard Ratio (95% CI)*
Total	40	100	8.4			138	100	28.9		
**Sex**										
Male	29	72.5	10.3	Reference	Reference	112	81.2	40.0	Reference	Reference
Female	11	27.5	5.6	0.55 (0.27, 1.10)	0.55 (0.28, 1.11)	26	18.8	13.3	0.34 (0.22, 0.51)	0.34 (0.22, 0.52)
**Age, years**										
≤ 60	10	25.0	5.7	0.56 (0.27, 1.15)	0.57 (0.28, 1.16)	31	22.5	17.5	0.49 (0.33, 0.73)	0.50 (0.33, 0.74)
> 60	30	75.0	10.0	Reference	Reference	107	77.5	35.6	Reference	Reference
**Race/Ethnicity**										
White (non-Hispanic)	37	92.5	8.5	Reference	Reference	133	96.4	30.6	Reference	Reference
Black (non-Hispanic)	0	0	0	N/A	N/A	0	0	N/A	N/A	N/A
Hispanic	1	2.5	10.7	1.26 (0.17, 9.14)	1.27 (0.17, 9.21)	3	2.2	32.1	1.05 (0.33, 3.30)	1.06 (0.34, 3.33)
Asian, Pacific Islander or American Indian/Alaska Native	1	2.5	12.5	1.47 (0.20, 10.8)	1.48 (0.20, 10.8)	1	0.7	12.5	0.41 (0.06, 2.93)	0.41 (0.06, 2.92)
Unknown	1	2.5	15.4	1.82 (0.25, 13.2)	1.86 (0.26, 13.6)	1	0.7	15.4	0.50 (0.07, 3.60)	0.53 (0.07, 3.78)
**Latitude, degree**										
≤ 35	19	47.5	8.8	Reference	Reference	71	51.4	32.9	Reference	Reference
> 35	21	52.5	8.0	0.91 (0.49, 1.70)	0.92 (0.49, 1.71)	67	48.6	25.6	0.78 (0.56, 1.09)	0.78 (0.56, 1.10)
**Average net erythemal exposure**										
≤ 170	25	62.5	7.5	Reference	Reference	91	65.9	27.5	Reference	Reference
> 170	15	37.5	10.3	1.37 (0.72, 2.59)	1.34 (0.71, 2.55)	47	34.1	32.3	1.17 (0.83, 1.67)	1.15 (0.81, 1.64)
**State of residence**										
California	16	40.0	10.9	Reference	Reference	41	29.7	28.0	Reference	Reference
Florida	9	22.5	8.9	0.82 (0.36, 1.84)	0.79 (0.35, 1.80)	36	26.1	35.8	1.28 (0.82, 1.99)	1.23 (0.79, 1.93)
Georgia	0	0	0	N/A	N/A	5	3.6	37.2	1.33 (0.52, 3.36)	1.38 (0.54, 3.49)
Louisiana	3	7.5	16.6	1.51 (0.44, 5.17)	1.53 (0.45, 5.26)	4	2.9	22.1	0.79 (0.28, 2.20)	0.81 (0.29, 2.25)
Michigan	3	7.5	12.3	1.11 (0.32, 3.82)	1.14 (0.33, 3.93)	2	1.5	8.2	0.29 (0.07, 1.21)	0.30 (0.07, 1.25)
North Carolina	3	7.5	7.6	0.69 (0.20, 2.36)	0.68 (0.20, 2.34)	13	9.4	32.9	1.17 (0.63, 2.19)	1.16 (0.62, 2.16)
New Jersey	3	7.5	4.9	0.45 (0.13, 1.54)	0.45 (0.13, 1.53)	18	13.0	29.9	1.06 (0.61, 1.84)	1.05 (0.60, 1.82)
Pennsylvania	3	7.5	4.1	0.37 (0.11, 1.27)	0.36 (0.11, 1.25)	19	13.8	25.9	0.92 (0.54, 1.59)	0.90 (0.52, 1.55)

* Adjusted for sex and age
